# Increasing land use drives changes in plant phylogenetic diversity and prevalence of specialists

**DOI:** 10.7717/peerj.1740

**Published:** 2016-03-01

**Authors:** Soraya Villalobos, Jana C. Vamosi

**Affiliations:** Department of Biological Sciences, University of Calgary, Calgary, Alberta, Canada

**Keywords:** Phylogenetic community structure, Functional diversity, Trait composition, Pollinator specialization

## Abstract

Increased human land use has resulted in the increased homogenization of biodiversity between sites, yet we lack sufficient indicators to predict which species decline and the consequence of their potential loss on ecosystem services. We used comparative phylogenetic analysis to (1) characterize how increasing conversion of forest and grasslands to grazing pasturelands changes plant diversity and composition; (2) examine how changes in land use relate to declines in functional trait diversity; and (3) specifically investigate how these changes in plant composition affect the prevalence of zygomorphy and the possible consequences that these changes may have on pollinator functional groups. As predicted, we found that the conversion to grazing pasturelands negatively impacted species richness and phylogenetic composition. Clades with significantly more represented taxa in grasslands (GL) were genera with a high representation of agricultural weeds, while the composition was biased towards clades of subalpine herbaceous wildflowers in Mixed Forest (MF). Changes in community composition and structure had strong effects on the prevalence of zygomorphic species likely driven by nitrogen-fixing abilities of certain clades with zygomorphic flowers (e.g., Fabaceae). Land conversion can thus have unexpected impacts on trait distributions relevant for the functioning of the community in other capacities (e.g., cascading effects to other trophic levels (i.e., pollinators). Finally, the combination of traits represented by the current composition of species in GL and MF might enhance the diagnostic value of productivity and ecosystem processes in the most eroded ecosystems.

## Introduction

Natural communities are currently facing extensive land use modifications, which have the potential to greatly alter species composition and structure (Smart et al., 2006; Watts et al., 2012). Farming, urbanization, and grazing decrease the available habitat for a large number of species, and usually allow persistence of only those species with adaptations for survival within modified ecosystems (Watts et al., 2012). The traits that determine whether a species can persist in a given habitat are likely those that allow survival under changing environmental conditions (Mouillot et al., 2013), yet this may have consequences on the prevalence of other traits that may affect ecosystem functioning. Despite the large effects of traits on ecosystem functioning, we are only beginning to understand how critical traits connect with land use changes (Clough et al., 2014).

With habitat modification, ecosystems face not only reduction of number of species, but also the loss of the ecological interactions that maintain ecosystem function (Wittmann et al., 2008; Letcher, 2010; Valiente-Banuet et al., 2014). Grasslands in particular have recently been noted as especially susceptible to land use changes (Weiner et al., 2011), with the effects on plant species composition having consequences to plant–pollinator mutualisms (Clough et al., 2014). In particular, emerging studies indicate the loss of more specialised species due to their highly vulnerability to habitat loss (Weiner et al., 2011; Dulvy et al., 2014).

In flowering plants, zygomorphy (i.e., bilateral symmetry) is though to serve as an adequate surrogate for functional group specialization of pollinators (Davila et al., 2012; Vamosi et al., 2014). The strong association between floral zygomorphy and insect pollinator accessibility is mostly promoted by the morphological restrictions that allow certain pollinator types to visit them (i.e., flies are generally not sufficiently versatile to visit restrictive flowers such as that presented by zygomorphic species; Vamosi et al., 2014). In this sense, flowers with bilateral symmetry are more ecologically specialized in their species-specific functional role (Eltonian’s perspective, Devictor et al., 2010): they establish a specialized pollinator system where pollinator assemblages are primarily shaped by size or tongue length (Gong & Huang, 2009). Previous studies have been equivocal with regard of the extent to which the functions that specialists perform in the ecosystem is insured by more generalist species (i.e., functional redundancy) (Bascompte, Jordano & Olesen, 2006; Cadotte, Cardinale & Oakley, 2008; Devictor et al., 2010). While some authors hypothesize that generalists provide a buffer for the current erosion of biodiversity (Costanza & Mageau, 1999; Scheffer et al., 2015), more recent studies suggests that species with a particular combination of traits may support more functions that cannot be supplied by species with more generalists habits (Manning et al., 2015; Walker, 2015).

Theoretical and experimental studies highlight that functional trait diversity can explain more variation in ecological processes that other types of biodiversity metrics (e.g., species richness) (Cadotte et al., 2009; Srivastava et al., 2012; Pellissier et al., 2014). Phylogenies are rapidly developing as a useful tool to summarize these functional traits (Cadotte et al., 2009; Faith, 2015), but are based on the assumption that closely related species are more ecologically similar than distantly related (i.e., share ecological roles). If there is a strong phylogenetic signal in the traits that determine ecosystem functioning (Flynn et al., 2011), patterns of phylogenetic diversity can be utilized to address key questions about functional differences related to ecosystem functions (i.e., biological, physical and geochemical processes that occur within an ecosystem).

The turnover and diversity of critical traits has been poorly studied in terms of land conversion. Understanding how disturbance differentially affects species and communities is a critical step towards prioritizing species and sites for conservation. Generally, specialist species are thought to be more susceptible to disturbance (Colles, Liow & Prinzing, 2009), in part due to their narrow range and relative rarity of occurrence. Although some studies have addressed the influence of land use intensity on rare species (Mouillot et al., 2013; Egorov et al., 2014; Mi et al., 2015), and rarity is often used as proxy for habitat specialisation, there are fewer studies that examine biotic specialization (Dorado et al., 2015). Zygomorphy, viewed as a functional trait, offers one trait that allows for some examination of how biotic specialization associates with disturbance because zygomorphic species allow visits from fewer functional groups of pollinators (Vamosi et al., 2014). Furthermore, we can quantify how a functional trait contributes to the overall phylogenetic community structure patterns if we detect a phylogenetic signal in its distribution within the assemblage (Mi et al., 2015) and predict how this might affect higher trophic levels.

For effective land management, we need to understand how restoration efforts will be impeded by trait changes produced by increases in land use intensity and forest fragmentation. We currently lack sufficient indicators to predict which species will be most affected and what consequence their potential loss will have on ecosystem services (Garnier & Navas, 2011; Cardinale et al., 2012). The identification of vulnerable clades, a major goal of conservation prioritization, is still largely based on trial and error due to the lack of comparative metrics that effectively capture the range of morphological and ecological diversity that species contain (Gossner et al., 2014). This study aims to the contribute to the identification of traits with functional properties that change in response to environmental conditions (Ricotta et al., 2015; Cadotte et al., 2015).

To better understand how trait diversity changes with land use, we characterize the effect of land conversion on reducing plant phylogenetic diversity and proportion of zygomorphic species (i.e., specialists) of plant communities in Southern Alberta.

Explicitly, we explore whether: (1) increasing land use (specifically, conversion of forest and grasslands to grazing pasturelands) is correlated with changes in plant species diversity and composition; (2) changes in land use relate to declines in phylogenetic diversity and community structure; and (3) declines in phylogenetic diversity relate to declines in diversity of a trait, such as zygomorphy, that potentially offers an indicator towards predicting the vulnerability of plant assemblages.

## Methods

### Plant community

We compiled a database of flowering plant communities using The Alberta Biodiversity Monitoring Institute (ABMI) vascular plant lists. ABMI has collected information about terrestrial ecosystems visiting approximately 330 sites each year, five times throughout spring and summer in the entire province of Alberta (http://www.abmi.ca). The survey for vascular plants was carried out delimiting a 1-hectare area surrounding the site centre and further dividing it into 50 × 50 m quadrants, where all plants were observed and collected at the site (http://www.abmi.ca/abmi/rawdata/rawdataselection.jsp). Using the 20 km National Forest Inventory (NFI) grid, 10 Eco-regions were defined based on the dominant plant community present or inferring that would have been present pre-disturbance. Taxonomical identification of plants was recorded *in situ* and vouchers of specimens deposited in the multiple collections in each site.

To date 769 different sites have been surveyed from 2003 to 2012 and 100% of the collection are taxonomically identified. Climatic variables (temperature and precipitation means) for collection dates in each collection sites were obtained from Environment Canada weather stations (http://climate.weather.gc.ca/prods_servs/cdn_climate_summary_e.html).

**Figure 1:**
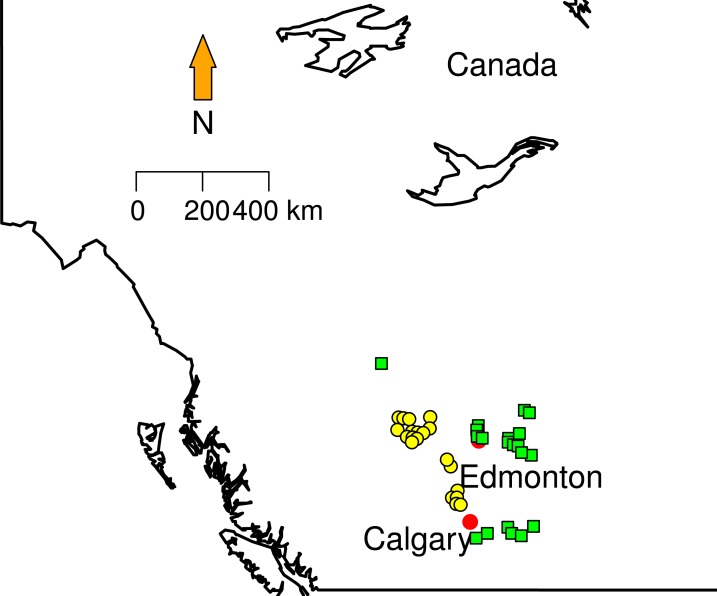
Sampling localities. Green squares correspond to Grassland (GL) communities and yellow triangles represent Mixed Forest (MF).

**Table 1:** Land conversion index. Normalized Rangeland Health Assessment (NRHA) score for each land cover site.

LCT	Site	NRHA
Grassland (GL)	706	0.4
	860	0.57
	861	0.48
	1,094	0.62
	1,442	0.35
	1,459	0.62
	1,481	0.45
	1,483	0.45
	1,496	0.57
	1,513	0.62
	983	0.45
	1,016	0.57
	1,022	0.4
	1,050	0.5
	1,051	0.45
	1,056	0.51
	1,057	0.39
	1,058	0.5
	1,092	0.6
	991	0.57
Mixed Forest (MF)	974	0.25
	1,001	0.24
	1,002	0.13
	1,003	0.13
	1,041	0.18
	1,068	0.13
	1,071	0.24
	1,072	0.13
	1,073	0.13
	1,103	0.13
	1,104	0.26
	1,105	0.24
	1,136	0.24
	1,239	0.13
	1,354	0.13
	1,378	0.18
	1,379	0.13
	1,403	0.13
	1,404	0.13
	1,208	0.13

### Land use

We randomly selected 20 sites in two different land cover classes area in Southern Alberta that clearly show conversion of forest to grazing pasturelands: Grassland and Mixed Forest (Fig. 1). Mixed Forest (MF) contains areas with at least a 10% crown closure of trees, where neither coniferous nor broadleaf trees account for 75% or more of crown closure. Grassland (GL) is characterized by native grasses (more than 20% ground cover), shrubs (less than 25%) or few trees (ABMI, 2014). Some proportions of this land cover type are affected by both human (e.g., cattle grazing, crops) and natural (e.g., flooding, fire) disturbances recorded from less than 5% to 100%. From these data each site in GL and MF was categorized in terms of the type and disturbance level. We used Rangeland Health Assessments RHA (Adams et al., 2009) as a proxy to measure land conversion (grazing levels). RHA establishes health categories that encapsulate natural function that ecosystems can perform (e.g., maintaining biological diversity, cycling nutrients and energy). The type of plants on the site, land cover layers, soil erosion levels, and the presence of invasive weed species were extracted from ABMI metadata. The Rangeland Health Assessment involves a maximum score of 100 divided in three categories, where 75 or greater indicates very healthy environment and less that 50 indicates unhealthy status. To better visualize the patterns; we created a new index normalizing RHA between 0 and 1 (Table 1). This index approaches zero with low land conversion levels and approaches 1 when land conversion is at maximum.

\begin{eqnarray*}\mathrm{RHAn =} \frac{N\mathrm{max}-n}{N\mathrm{max}} . \end{eqnarray*}

Here, *N*max is the maximum RHA score (100), and n is the observed RHA per site.

### Phylogenetic community structure and trait distribution

We generated a regional plant phylogeny using the online software Phylomatic (Webb & Donoghue, 2005) for all plant species in the dataset. The tree contains 896 species for which we have community occurrence data. To time-calibrate the branch lengths on the phylogeny, we applied ‘wikstrom’ ages to internal nodes (Wikstrom, Savolainen & Chase, 2001) with the BLADJ algorithm. Phylomatic provides only a rough approximation of the phylogeny, which could substantially affect our results, specifically in the estimation of phylogenetic signal (Davies et al., 2012). We use metrics that are robust to phylogenetic signal however (MPD and MNTD), such that estimates of branch lengths would not greatly change the qualitative results on phylogenetic structure (Cadotte, 2015).

For floral symmetry we hypothesized that the increasing land use reduces the presence of specialist (i.e., zygomorphic) plant species, selecting species with more generalist (i.e., actinomorphic) habits. The phylogenetic signal in zygomorphy was estimated within GL and MF with Blomberg’s K statistic (Blomberg, Garland & Ives, 2003), using the function Kcal in ‘picante’ version 1.6-2, R package (Kembel, 2009). *K* values close to zero indicate that a trait exhibits a random distribution on the phylogeny, while *K* values greater than 1 indicate a strong phylogenetic signal. The significance level was assessed by comparing observed branch length distance between species with the trait to a null model of shuffling taxa labels across the tips of phylogeny (Blomberg, Garland & Ives, 2003).

To determine which clades contribute disproportionately to the phylogenetic structure in each type of land cover, we used Adonis test and *nodesig* algorithm from Phylocom software (Webb, Ackerly & Kembel, 2008). An Adonis analysis examines whether the land cover type explains phylogenetic and taxonomic differences in GL and MF and *Nodesig* tests whether the species richness that coexists in a local community is represented in a particular clade with more or less frequency than expected from a null model. The model that we used gives the same chance of a species in the regional phylogeny to be included in the null model by randomly drawing the species composition (identity of species) and maintaining the species richness of each sample (Webb, Ackerly & Kembel, 2011). The procedure identifies the clades with significant overrepresentation (sigmore) or less representation of taxa (sigless) for each local community. Because vegetation type is known to harbour dramatically different plant species (e.g., trees clearly disproportionally inhabit MF, whereas herbs disproportionally inhabit GL), we applied *nodesig* for both Grassland and Mixed Forest using the pool of species present in sites of these lands covers types. Likewise, we implemented *nodesig* to test how land use conversion (LUC) affects plant composition for the highly disturbed communities (LUC scores under 0.5) and the most protected communities (LUC scores above 0.51).

The phylogenetic structure was calculated using Mean Pairwise Distance (MPD) and Mean Nearest Taxon Distance (MNTD) in the package vegan in R version 3.0.2 (Oksanen et al., 2013; R Core Team, 2013). Standardized effect sizes were calculated for MPD and MNTD by comparing the observed phylogenetic relatedness to a null model generated by using the option ‘phylogeny shuffle’ that randomizes phylogenetic relationships among species (Webb, 2000).

We used multiple linear regression to analyze the relationship between plant phylogenetic diversity, land conversion and climatic variables (temperature and precipitation) for all species, as well as for the group of zygomorphic present in each plant community. The land cover type was used as a factor to assess how phylogenetic diversity change across land conversion. To evaluate whether the land use conversion has different effects in the different land cover types, we compare the regression lines with an ANCOVA by testing the significance of the interaction Land Cover Type (LCT) × Land Use Conversion (LUC).

## Results

### Species composition

Without taking phylogenetic relationships into account, the most protected sites (LUC 0–0.5) hosted higher average total species richness and more zygomorphic species (39.8 species and 6.9 zygomorphic species) than the most land converted sites (20.3 species and 3.7 zygomorphic species). These differences were significant in terms of the total species richness (*t* = 5.407, *df* = 10, *p*-value = 0.0003) and the richness of zygomorphic species (*t* = 2.66, *df* = 6, *p*-value = 0.02) between plant communities with low and high LUC index (Table 1). Likewise the proportion of zygomorphic species increased with land use conversion (*χ*^2^ = 0.08, *df* = 1, *P* > 0.05).

As predicted, the taxonomic and phylogenetic composition between the two land cover types was significantly different (Adonis analysis: F1, 38 = 10.099; *r*2 = 0.20997; *p* = 0.001; F1, 38 = 1.8302; *r*2 = 0.04595; *p* = 0.001). Positive spatial autocorrelation was observed in plant species composition (Monte-Carlo Mantel test; Observation: 0.16; *p* = 0.00057).

### Phylogenetic community structure and land conversion

**Figure 2:**
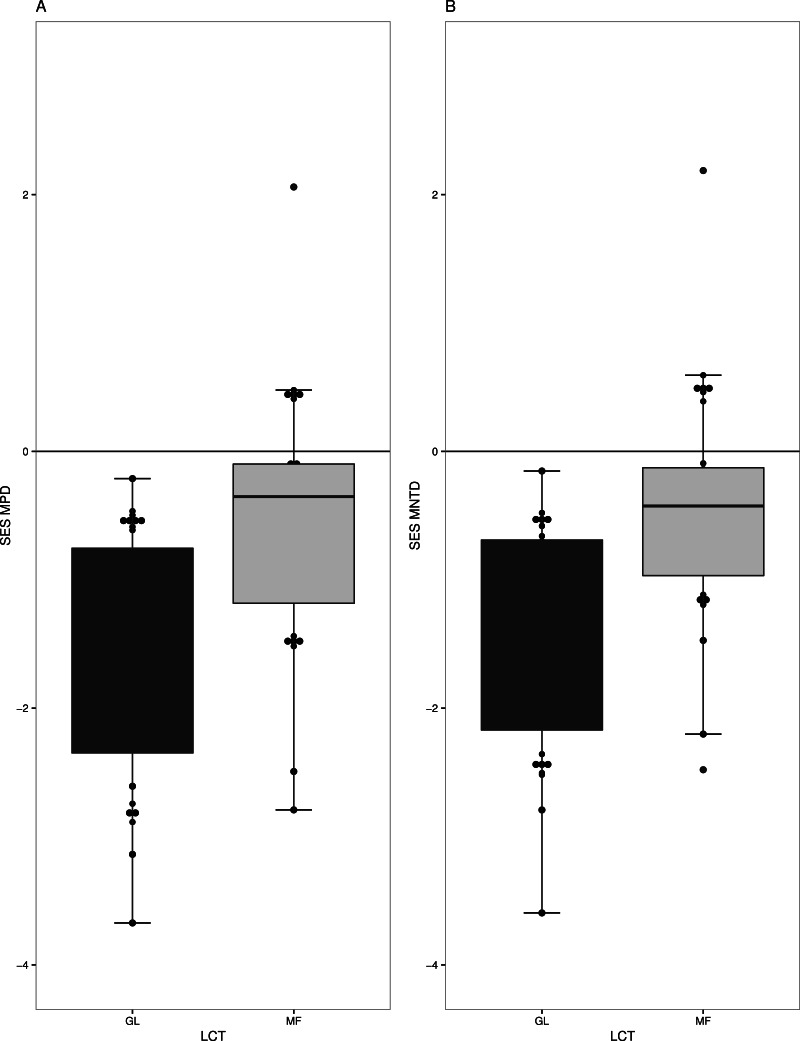
Measures of phylogenetic community structure for two land cover types (LCT), Grassland (GL) and Mixed Forest (MF). GL and MF communities have phylogenetic clustering in species when (A) phylogenetic diversity was compared to that expected from a null phylogeny, SES MPD (*P* < 0.05), and (B) when accounted on the terminal phylogenetic composition of communities, SES MNTD (*P* < 0.05).

Overall, we found strong phylogenetic signal for zygomorphy in our regional phylogeny (*K* = 1.408 *p*-value = 0.006). Forest (MF) and grasslands (GL) had very different levels of phylogenetic structure in their respective plant communities. GL communities generally have phylogenetic clustering in species (*t* = − 6.5536, *df* = 18, *p*-value =3.70^−06^) (Fig. 2A), especially when only the subset of zygomorphic species was examined (−2.44 *df* = 18, *p*-value = 0.02). Similarly, when account on the terminal phylogenetic composition of communities (mean nearest taxon diversity MNTD), GL and MF showed patterns of significant clustering (*t* = − 6.80, *df* = 18, *p*-value =2.26^−06^, *t* = − 2.34, *df* = 18, *p*-value = 0.03) (Fig. 2B).

**Figure 3:**
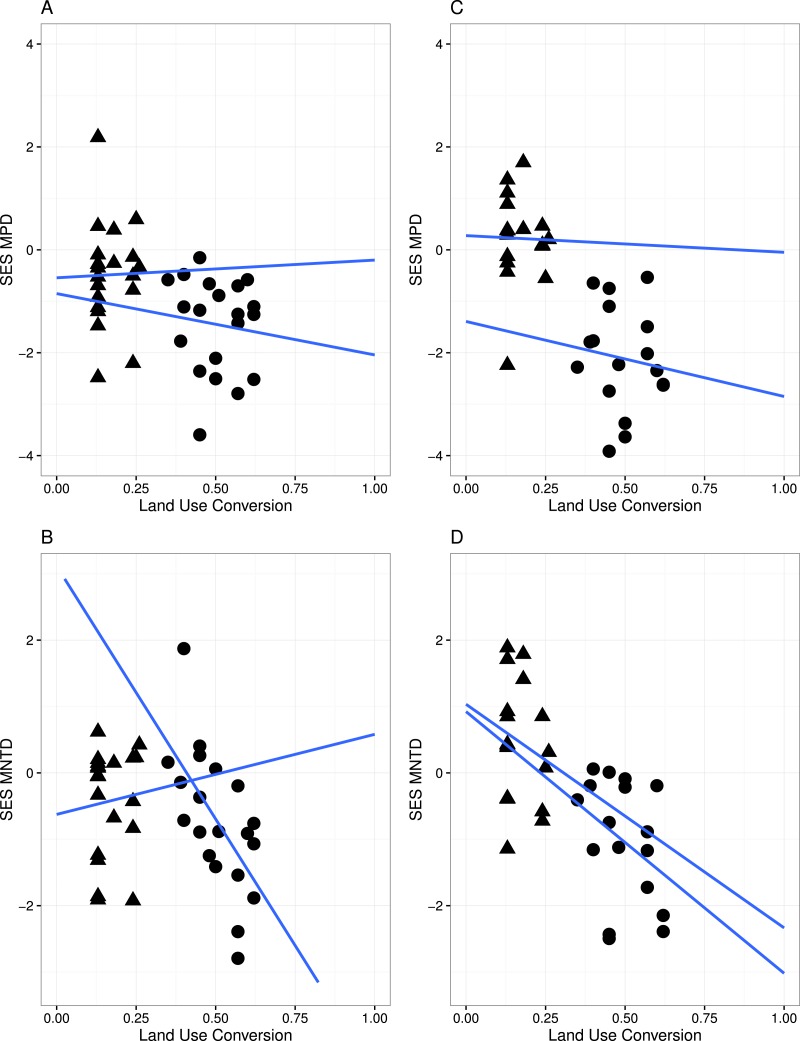
Linear regression with regression slopes between mean pairwise distance (MPD), mean nearest taxon distance (MNTD) and land conversion index for all species and zygomorphic species. Triangles, MF sites; circles, GL plant communities. (A–B) represent analyses where all species in each communities are considered; (C–D) represent analyses where only zygomorphic species in each communities are considered. The blue lines are the fit lines spanning the full range of the plot for the two set of data.

A significant relationship between the variables in the linear regression model (Fig. 3) indicated that land conversion has a strong negative effect on MPD for all species in the community (*r* = − 4.565, *P* < 0.05) as well as for zygomorphic assemblages (*r* = − 6.279, *P* < 0.05). Zygomorphic species MNTD significantly decreases with land use conversion (*r* = − 5.197, *p* < 0.05), while the non-significant LCT × LUC interaction (Table 2) indicated that the rate of MPD and MNTD decline with land conversion did not depend on the different land cover types for all species and zygomorphic species. Likewise, the rate of MPD and MNTD decline were not explained by the variation in temperature and precipitation between the two vegetation types (Table 2).

### Phylogenetic compositional changes

**Figure 4:**
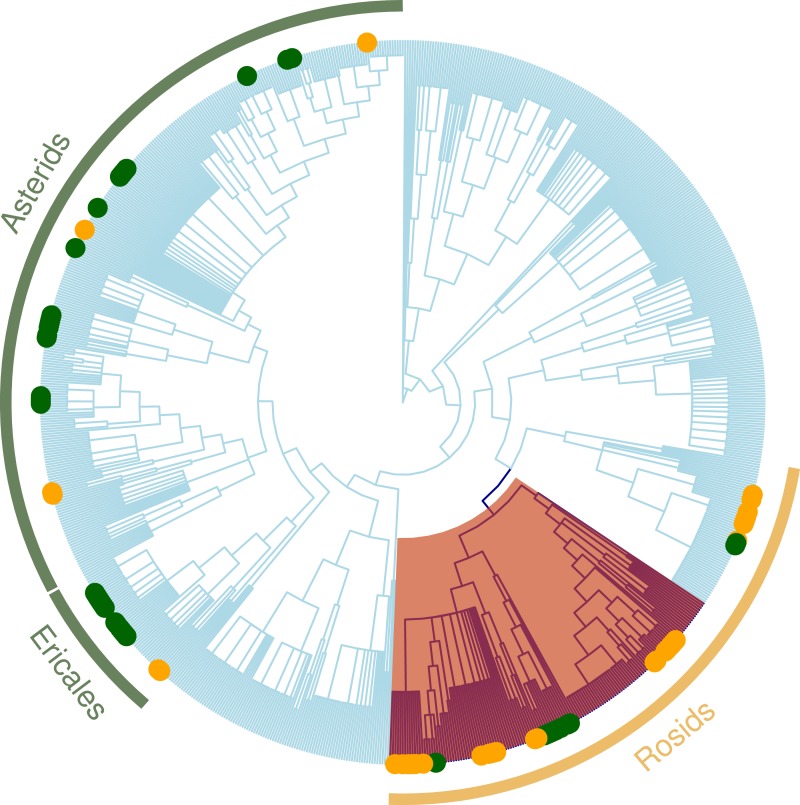
Regional plant phylogeny with 891 species. Green circles correspond to species of the important clades in less altered communities (LUC < 0.05) and yellow circles represents important clades in highly altered communities (LUC > 0.05; see Table 4). Red shading band indicates nitrogen-fixing clade within Rosids, which we speculate is an important factor contributing to why these clades are significantly more represented in the more disturbed areas. Green bands correspond to Ericales and Asterids clades that were more represented in the more protected zones.

The *nodesig* analysis indicated that disproportionately represented clades in GL plant communities were clades of predominantly ruderal species and those with berries (*Trifolium*, *Melilotus* and *Rubus*) (Table 3 and Fig. 4). Likewise, clades that significantly contribute to the phylogenetic structure in Mixed Forest are mostly clades of subalpine herbaceous wildflowers. Overrepresented clades correspond to “wintergreen” berries with shaded moist wood habit (Asparagales, Ochidaceae, Ericaceae) (Table 3). Asteraceae were significantly underrepresented in the MF plant community.

When we tested how LUC index affects clade composition (Table 4), the *nodesig* analysis indicated that clades with significantly more represented taxa for the more disturbed areas (LUC scores above 0.5) were nitrogen fixers (*Trifolium* and *Medicago*) (Fig. 4). For the most protected sites (LUC score below 0.5) Caprifoliaceae and Eri*c* aceae (families of wild flowers and berries) and the genus *Hieracium* (Asteraceae) were significantly more represented. Monocots and were less represented in these sites.

**Table 2:** Effect of climatic variables, land cover type, land use conversion and its interaction on phylogenetic diversity (A) MPD (mean pairwise distance) and (B) MNTD (mean nearest taxon distance).

A		MPD (all plants)		MPD (zygomorphic)	
	*df*	*F*	*P*	*F*	*P*
Land Cover Type (LCT)	1	0.740	0.3911	43.656	2.5e^−07^^*^
Land Use Conversion (LUC)	1	8.858	0.0051^*^	5.39	0.0272^*^
LCT × LUC	1	0.093	0.7626	0.04	0.8344
Precipitation	1	2.663	0.111	3.241	0.082
Temperature	1	0.483	0.491	0.001	0.977
Residuals	36			30	

B		MNTD (all plants)		MNTD (zygomorphic)	
	*df*	*F*	*P*	*F*	*P*
Land Cover Type (LCT)	1	4.231	0.047^*^	2.5425	2.07e^−0.5^^*^
Land Use Conversion (LUC)	1	4.109	0.050	0.109	0.744
LCT × LUC	1	4.187	0.048^*^	0.013	0.909
Precipitation	1	2.438	0.129	2.50	0.145
Temperature	1	0.313	0.580	0.321	0.576
Residuals	36				

**Notes.**

* Significant value *P* < 0.05

**Table 3:** Clades significantly contributing to the community structure in Grassland and Mixed Forest plant community (Phylocom procedure *nodesig*; Webb, Ackerly & Kembel, 2008).

Land cover type	Sig more/less	Node	Family	Clade
Grassland	More	Dipsacales		Euasterids II
	More	Rosales		Eudicots
	More	Rosaceae		Eudicots
	More	*Rubus*		Eudicots
	More	*Trifolium*	Fabaceae	Eudicots
	More	*Melilotus*	Fabaceae	Eudicots
Mixed Forest	More	Asparagales		Monocots
	More	Orchidaceae		Monocots
	More	Ericales		Basal Asterid
	More	Ericaceae		Basal Asterid
	More	*Pyrola*		Basal Asterid
	More	Rosales		Eudicots
	More	*Rubus*	Rosaceae	Eudicots
	Less	Asterales		Euasterids II
	Less	Asteraceae		Euasterids II

**Table 4:** Clades composition with low and high land use conversion (LUC) score (Phylocom procedure *nodesig*; Webb, Ackerly & Kembel, 2008).

Land use conversion	Sig more/less	Node	Family	Clade
LUC score under 0.5	More	Dipsacales		Euasterids II
	More	Caprifoliaceae		Asterids
	More	Ericales		Basal Asterid
	More	Ericaceae		Basal Asterid
	More	Malpighiales		Rosid
	More	*Hieracium*	Asteraceae	Asterids
	Less	Monocots		
LUC Score above 0.51	More	*Medicago*	Fabaceae	Eudicots
	More	*Trifolium*	Fabaceae	Eudicots

## Discussion

The loss of species from native communities represents a loss of functional traits that contribute to the maintenance of ecosystem function. Recent studies have found that some groups of specialists can occur at high diversity in systems with intensive variation of land use (Allan et al., 2014; Allan et al., 2015). We studied sites with different original vegetation types and degrees of disturbances and found that, overall; land conversion has a strong negative effect on the phylogenetic structure of communities, and, in particular, narrowed the phylogenetic distribution of zygomorphic species. While it may not be surprising that land use conversion has a significant influence on the reduction of plant diversity, our study highlights a substantial modification in the trait composition of the plant community (Perrings et al., 2011; Worm et al., 2015) as well as the phylogenetic underpinnings of these trait changes. Identification of the particular clades with non-random patterns of phylogenetic structure indicate that the changes in the prevalence of zygomorphy are possibly driven by habitat filtering for nitrogen-fixing species, where zygomorphic flowers happen to be prevalent (e.g., Fabaceae; Fig. 4). Land conversion can thus have unexpected impacts on trait distributions relevant for the functioning of the community in other capacities (e.g., maintaining healthy populations of pollinators).

Our analysis of clade distributions identified Ericaceae, Orchidacece and *Hieracium* (Asterids) overrepresented in sites with low conversion, whereas Rosaceae and, in particular, *Rubus* (Rosids), were more presented in areas with high conversion indices. The latter finding is surprising given that, in other studies, these clades have been suggested as good indicators of low land use intensity (Manning et al., 2015). We posit that the significant phylogenetic clustering versus randomness found in the two lands cover types is driven by traits that are represented disproportionately in these clades due to their influence on survival, growth and reproduction under differing conditions (Kembel, 2009). Low MPD in the more converted zones may suggest that disturbance promotes the selection of close relatives with similar environmental requirements (Webb et al., 2002). In our system, the overrepresentation of members of the nitrogen-fixing clade in the rosids (Werner et al., 2014) in most disturbed sites is likely indicative of the important functional role of nitrogen-fixing in highly converted sites, with any associated traits that are predominant in this clade effectively hitchhiking to greater prevalence.

That we found parallel patterns of increased phylogenetic clustering with disturbance in our zygomorphic subset suggests that zygomorphy was not the key trait that habitat selection was acting upon, yet would potentially have consequences for downstream habitat selection of certain pollinator functional groups (e.g., flies cannot readily forage on zygomorphic flowers). Clearly, zygomorphy is not the only floral trait that restricts certain functional groups of pollinators. For example, many species of Caprifoliaceae have tubular actinomorphic flowers that also restrict short-tongued flies from visiting and species in this clade were restricted to low LUC sites (see Table 4). Thus, while an understanding of phylogeny and traits can allow us to better predict how communities will change with disturbance, future analyses will require more complete knowledge of how a number of traits change, as well as the downstream consequences of these changes.

Through an understanding of traits, we can begin to predict how these compositional changes might affect other aspects of ecosystem functioning. The decrease in phylogenetic diversity observed in disturbed GL and MF sites points out that, upon disturbance, flowering plant communities are offering pollinators increasingly similar suites of floral resources (Blomberg, Garland & Ives, 2003). We found this to be true even when only the subset of zygomorphic species were analysed. Hence, the ecological functions that functionally diverse communities perform, including those unmeasured traits for which the phylogeny account for (e.g., hosting diverse pollinator communities) (Cadotte, Cardinale & Oakley, 2008; Flynn et al., 2011), would not being provided by the narrow phylogenetic breadth of the disturbed assemblages. In other words, like observed in Allan et al. (2014) and Allan et al. (2015), some specialist species thrive in disturbed environments. However, the potential consequence of these changes in composition is that, for these two particular types of plant communities, the reduction to a community of specialists at the plant trophic level, is that the combination of functional traits (i.e., functional redundancy) are likely reduced, which may cascade into a lower phylogenetic diversity of pollinators supported (Adderley & Vamosi, 2015).

While we do not find evidence that functional groups specialists (measured with a coarse surrogate) are particularly vulnerable to disturbance, the loss of generalists may have its own consequences. The loss of species with unique combination of traits will likely cause high impact on ecosystem processes (Olesen & Jordano, 2002; Mouillot et al., 2013). If zygomorphic species support fewer functional groups of pollinators, then phylogenetic diversity represents functional differences relevant for ecosystem functioning (Flynn et al., 2011), because increasing phylogenetic diversity translates into plant communities with combinations of traits that support a wide breath of pollinators, including flies that are often restricted from visiting zygomorphic flowers (Vamosi et al., 2014). Whether this individual trait can be used to capture the functioning of ecosystems in other aspects will require further experimental tests to determine the extent to which this specific trait represents an indicator of other ecosystem processes (e.g., contribution to the seed set production, total biomass). Floral morphological barriers that bear direct relationship with pollination and seed set production may enhance the diagnostic value of productivity if we quantify the flux of multiple services from the plant to the ecosystem (Garnier & Navas, 2011). Therefore, ecosystem processes could be estimated from this relevant (and readily identifiable) trait.

Our study uncovered no significant differences in the responses with land use between community types (MF vs. GL) suggesting that the effect of disturbance is not context dependent (although GL sites typically had higher levels of LUC than ML sites). Our findings in contrast with previous observational and experimental studies thus far, likely influenced by differences in sample size, historical land use record and taxonomic scales (Newbold et al., 2012; Egorov et al., 2014; Pellissier et al., 2014). For example, grasslands are host to extremely high local species richness, yet have lower betadiversity than forests (Wilson et al., 2012). Thus, conversion of forest to pasturelands is not necessarily correlated with declines in local plant species diversity (Egorov et al., 2014) yet our study indicates will experience greater changes in phylogenetic and functional composition. Future studies should examine more traits separately in order to establish which specific traits or combination of them may increase the diversity of the more eroded zones.

In summary, although it has been hypothesized that species poor communities may be composed of distantly related species (Helmus et al., 2010), we find more clustered assemblages in the more disturbed (and species-poor) sites. Dissecting the compositional changes involved with changes in species richness reveal that having closely related species could be a result of an increased representation of angiosperm clades that benefit in poor soils (the nitrogen fixing clade in Rosids in particular). This shift in community composition has downstream consequences in that this clade is represented by many zygomorphic species, that are largely floral resources for the bee functional group of pollinators and excludes many less versatile pollinators (Vamosi et al., 2014). While specialist plant species may be beneficial for bees, this composition shift may still result in an accelerated loss of species. Establishment of plant communities with high trait diversity in floral traits should be a consideration for rapid restoration of the converted landscapes.

## Conclusions

The changes in plant diversity with land conversion reinforce the idea that disturbance has strong effect in the reduction of functional composition. Overall, our findings suggest that changes in plant community composition favour the persistence of clades that may enhance soil conditions in the more degraded zones. Interestingly, this alteration in community composition increases the prevalence of zygomorphic clades, which would select for more versatile pollinators (such as bees) in a potential cascade of effects to other trophic levels. Considering recent concerns for bees and other pollinators (Kerr et al., 2015), understanding the causes of changes to floral resource diversity will become a topic of increasing importance.

## Supplemental Information

10.7717/peerj.1740/supp-1 Supplemental Information 1 Regional plant phyogenetic tree_data

10.7717/peerj.1740/supp-2 Supplemental Information 2 Plant abundance matrix_ data

## Additional Information and Declarations

### Data Availability

The following information was supplied regarding data availability: Raw data are provided as Supplemental Information.
